# Automated oscillometric blood pressure and pulse-wave acquisition for evaluation of vascular stiffness in atherosclerosis

**DOI:** 10.1007/s00392-017-1080-7

**Published:** 2017-02-06

**Authors:** Alexander Massmann, Jennifer Stemler, Peter Fries, Reinhard Kubale, Lutz Erwin Kraushaar, Arno Buecker

**Affiliations:** 1 Clinic of Diagnostic and Interventional Radiology, Saarland University Medical Center, Kirrberger Straße, Geb. 50.1, 66421 Homburg/saar, Germany; 2 adiphea GmbH, 35510 Butzbach, Germany

**Keywords:** Atherosclerosis, Ankle-brachial index, Arterial stiffness, Vascular resistance, Flow-mediated dilation

## Abstract

**Objective:**

Evaluation of diagnostic accuracy of an oscillometry-based device (VascAssist) combining fully automated ankle-brachial index (ABI) and pulse-wave velocity (PWV) assessment for detection of peripheral arterial disease (PAD).

**Subjects and methods:**

110 consecutive subjects including symptomatic PAD patients (*n* = 41) and healthy PAD-free participants (*n* = 69) were recruited. All subjects underwent standard manual Doppler-based ABI (sABI) and oscillometry-based automated ABI (aABI) measurements (VascAssist). Oscillometry by the VascAssist included central and peripheral PWV assessment. Additionally, arterial stiffness (AS) was evaluated by flow-mediated vasodilation (FMD) of the brachial artery in all patients. All symptomatic PAD patients underwent catheter angiography for endovascular intervention and post-interventional acquisition of sABI, aABI, PWV and FMD.

**Results:**

Sensitivity, specificity, PPV and NPV of aABI for detecting PAD was 73%, 100%, 100%, and 86% as compared to 80%, 96%, 92%, and 89% for sABI. Pearson-correlation for diabetics was *r* = 0.81; (*P* < .001) and for non-diabetics *r* = 0.77; (*P* < .001). Bland–Altman-analysis revealed a difference (95% CI) for diabetics of 0.09 (−0.22–0.4] and non-diabetics 0.022 [−0.25–0.295]. Weak correlation exists for FMD/AS analysis (pre-interventional *R* = 0.386, *P* = .043; post-interventional *R* = −0.06; *P* = .76) and significant increase of pre-/post-interventional PWV analysis (*P* < .001).

**Conclusion:**

Combined automatic ABI and PWV acquisition with the VascAssist device showed excellent diagnostic accuracy for detection of PAD. Compared to FMD, AS analysis may serve as an investigator-independent (screening) tool for determination of functional vascular damage in atherosclerosis.

## Introduction

Atherosclerosis is the major reason for cardiovascular-related death in western countries. The disease is mostly asymptomatic at an early stage. Screening for peripheral artery disease (PAD) is usually made by non-invasive measurement of ankle-brachial index (ABI) [1–3]. In contrast to usual manual ABI assessment, automated assessment is more efficient [4] and less time-consuming without the need for specific training [5, 6]. Unfortunately, statistical evaluation of validity and reliability between automated and standard ABI assessment in previous studies was often imprecise [7].

Early detection of initial vascular damage is achievable by assessment of endothelial function [8]. In addition to ABI, non-invasive flow-mediated dilatation (FMD) is an early marker for atherosclerosis, and has been identified as a predictor for potential cardiovascular events [9]. Despite a high level of standardization, the time-consuming measuring of FMD highly depends on an operator’s expertise, necessitates patient’s compliance and is altered by a number of environmental variables [10].

Moreover, arterial stiffness is evaluable by pulse-wave analysis (PWA) of aortic/brachial pulse-wave velocity (PWV). Increased aortic PWV, augmentation index (AUI), and decreased arterial elasticity are typically present in patients with PAD [11, 12]. These findings are clearly identified as independent predictors for cardiovascular-disease-related morbidity and mortality [13–15]. Decreased small artery elasticity and increased AUI are associated with pathologic ABI [16, 17], which is the most important predictor for PAD and cardiovascular risk [18].

The beforehand mentioned diagnostic tests use different approaches to assess arterial distensibility as a surrogate for vascular function. Reduced distensibility, or arterial stiffness (AS) can essentially be expressed by the relationship between aortic compliance and total peripheral arterial resistance, which are derived from PWV analysis of oscillometrically acquired blood pressure curves [19].

The purpose of our study was to validate the diagnostic accuracy of a dedicated oscillometric automated device (VascAssist) in patients with known PAD and healthy volunteers. The primary objective was to compare manual ankle-brachial index (sABI) measurement using Doppler-ultrasound and mercury-sphygmomanometry as the reference standard vs. investigator-independent automated ABI (aABI) measurement. Secondary objectives included AS compared to (pre- and post-interventional) sABI, aABI and FMD.

## Materials and methods

Overall, 110 subjects were consecutively recruited including 41 patients [female 15%, mean age 69.0 ± standard deviation 11.4 (range 40–94) years, *n* = 20 diabetics] with symptomatic PAD and impaired walking capacity <200 m (intervention-group), and 69 voluntary participants (control group) presumably free of PAD (53% female, 46.5 ± 15.2 [22–75] years, 4 diabetics). Table 1 shows demographic data of intervention and control group. Consistency of different groups is mandatory to exclude confounding. However, the aim of our study was to analyze the effectiveness and discriminatory power of sABI and aABI in a healthy and diseased population, separately. The diagnostic accuracy study was conducted in conformance with the Declaration of Helsinki, approved by the local ethics committee (Ethikkommission des Saarlandes, Germany) and registered with the German Registry for Clinical Studies (DRKS) Trial No. DRKS00005777. Each participant provided written informed consent. Demographic information was acquired after consent was obtained.

**Table 1:** Demographic data of study population

Characteristics	Symptomatic PAD *n* = 41	PAD-free *n* = 69	*p* value
Age (years)	69.0 ± 11.4 [40–94]	46.5 ± 15.2 [22–75]	<0.0001
Female sex	17.1%	56.5%	<0.0001
Postmenopausal	100%	46.2%	0.0112
BMI (kg/m^2^)	27.5 ± 5.2 [18.4–41.8]	25.6 ± 4.3 [18.3–38.6]	ns (0.060)
Diabetes mellitus	48.8%	5.8%	<0.0001
With Metformin	50%	1.5%	–
With Insulin	50%	0%	–
Both	5%	0%	–
Smoker (pack years)	43.9% (53 ± 27 [25–150])	14.5% (16 ± 12 [1–35])	0.0012
Ex-smoker (years)	41.5% (16 ± 16 [1–50])	20.3% (14 ± 10 [1.5–30])	0.0274
Coronary heart disease	34.2%	0%	<0.0001
Hypertension	73.2%	17.4%	<0.0001
Dyslipidemia	56.1%	20.3%	0.0002
Regular alcohol intake	9.8%	0%	<0.0001

Exclusion criteria included at least one of the following: participation in another study, malignancy, cardiac pacemaker, limb surgery, current or pregnancy <12 months, necrosis at any measurement location, axillary lymphadenectomy, convulsions, spasms, tremor of any kind.

Individuals in the *intervention-group* were characterized by symptomatic PAD with an impaired walking capacity <200 m. All these patients had been consecutively referred to our angiography department for transfemoral catheter angiography combined with endovascular interventions. ABI, AS, and FMD measurements were performed before and after angiography. Interventional procedures (*n* = 44) included iliac stenting (*n* = 10), femoro-popliteal (*n* = 25) balloon-angioplasty or stenting, or endovascular aortic repair EVAR (*n* = 9). Three patients underwent combined procedures.

The *control group* encompassed PAD-free participants with a non-compromised, normal walking capacity. All these participants underwent only non-invasive ABI, AS, and FMD measurements.

In both groups, all measurements were performed in the morning by the same experienced investigator in a temperature-controlled room (24 ± 1 °C) with the subject in a supine position, and after having rested supine for 15 min prior to measurements.

### Comparison of sensitivity between ABI measurement methods

#### ABI measurements

Appropriately sized sphygmomanometric cuffs were used for sABI and aABI measurements. The participants rested supine for 15 min before pressure measurements were obtained.

Measurements for sABI were performed with a commercially available unidirectional Doppler device (handydop, ELCAT, Wolfratshausen, Germany). A brachial pneumatic cuff was applied to the left upper arm, inflated to supra-systolic pressure and deflated slowly until a Doppler-flow signal was detected. Determination of ankle pressure was determined similarly at both ankles with flow detection over the dorsal pedal and posterior tibial arteries. ABI was calculated as the higher of the two pedal pressures divided by the arm pressure. Measurements were taken in triplicate.

Measurements for aABI were also performed in triplicate, immediately following the sABI measurements, using the VascAssist device (VA) (iSYMED GmbH, Butzbach, Germany). Four pneumatic cuffs were applied to both arms and distal lower legs. aABI was calculated for each extremity as the relevant ankle pressure divided by the higher of the two arm pressures.

### Investigation of arterial stiffness index in comparison to FMD and ABI

#### FMD measurements

**Fig. 1:**
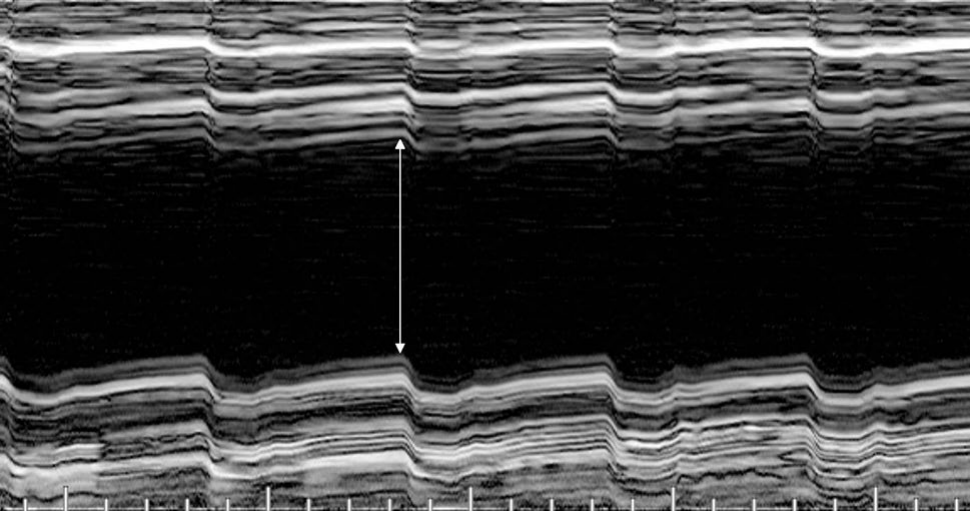
Ultrasound in M-mode (Acuson S2000 @18 MHz 18L6 40 frames/s, Siemens Medical, Erlangen, Germany) illustrates cross-sectional imaging of the brachial artery. The vessel diameter is determined according to the orthogonal distance between the intima reflexion zone (*double arrow* = 4.9 mm; longitudinal scale 25 mm)

The FMD measurements were performed with patients in the supine position. Patients were instructed to avoid caffeine-, nicotine- and alcohol-containing products for at least 12 h before measurements. A blood pressure cuff was placed around the upper arm distal to the brachial artery segment that was explored. Ultrasound images were acquired using a commercially available ultrasound system with an 18 MHz high-resolution linear-array transducer (Acuson S2000, Siemens Healthcare, Erlangen, Germany). The cuff was continuously inflated 50 mmHg above the patient’s systolic blood pressure for 5 min. Brachial artery diameter and flow velocity were recorded using 2D echography prior to cuff inflation, at deflation and after deflation at 1 min intervals for 5 min. The probe was angulated at 90° for optimal morphologic B-mode imaging and <60° for optimal velocity acquisition. Diameter measurements were made with electronic calipers at the end of ventricular diastole (Fig. 1).

### Measurement of arterial stiffness index (AS)

For AS derivation, oscillometric blood pressure recordings were taken at the brachial and radial arteries of each arm in triplicate, subsequent to the FMD measurements. Pulse pressure curves were acquired at 1 kHz frequency. Proprietary PWA analysis algorithms were applied offline with the VascViewer software for Windows (iSYMED GmbH, Butzbach, Germany).

### Statistical analyses

Analyses were performed offline, using STATA 11 software (StataCorp, Texas, USA) by an investigator blinded to test conditions and patient identity. Demographic variables are presented as mean ± standard deviation and computed measures as mean ± standard deviation; categorical variables (i.e., diabetes status or sex) are presented as absolute frequencies and percentages.

A power analysis was conducted before recruitment to determine the appropriate number of study subjects. With significance set at *P* ≤ = 0.05, and assuming a correlation coefficient at *r* ≥ 0.3, 110 subjects provide a power of ≥0.89. Discrepancy of subject number in control and intervention group is based on the level of significance.

Correlation between measures was determined by linear regression. Agreement between aABI and sABI measurements was investigated using the Bland–Altman method [20].

Pearson’s correlations were calculated between each ABI measurement mode, and to evaluate correlations between FMD and AS measurements. Paired samples’ *t* tests were conducted to analyze pre-intervention vs. post-intervention differences in AS. Shapiro–Wilk tests were performed to determine normality of differences between both measures. Since sABI was derived using left arm blood pressure values only, aABI was recalculated (using left brachial pressure values only) accordingly for statistical analyses. To avoid the effects of interrelatedness of measures, within-subject ABI values were entered as independent observations for each leg.

Selection of ABI measurements for statistical analyses: The VascAssist software (VascViewer) alerts the investigator to potential invalidity of individual pressure recordings based on a plausibility assessment of the detection of (a) end-diastolic nadir, (b) systolic peak pressure and (c) pulsatility of the ankle pressure curves.

## Results

Overall, 278 ABI measurements were obtained in 110 participants. Twenty-eight measurements were excluded: In nine instances, ABI acquisition was impossible for aABI and possible for sABI. In 11 instances, ABI acquisition was possible for aABI and not possible for sABI. Furthermore, in eight instances, ABI acquisition was neither possible for aABI nor for sABI. The reason for technical failures of ABI determination is speculative. Severe (media-)sclerosis and vessel occlusion should be the most probable assumption.

### Comparison of sensitivity between ABI measurement methods

The number of patients misclassified as healthy was 11 (26.8%) vs. 8 (19.5%) for aABI vs. sABI, respectively, which constitutes a non-significant difference at *z* = 1.18 (*P* = .24). None of the healthy controls (*n* = 69) was misclassified by aABI vs. 3 misclassifications (4.3%) by sABI, which constitutes a non-significant difference at *z* = 1.69 (*P* = .09). Expressed as sensitivity, specificity, positive predictive power and negative predictive power, the aABI method scored at 73, 100, 100, and 86%, respectively, whereas the sABI method had corresponding values of 80, 96, 92, and 89%, respectively.

### Level of agreement between sABI and aABI

**Fig. 2:**
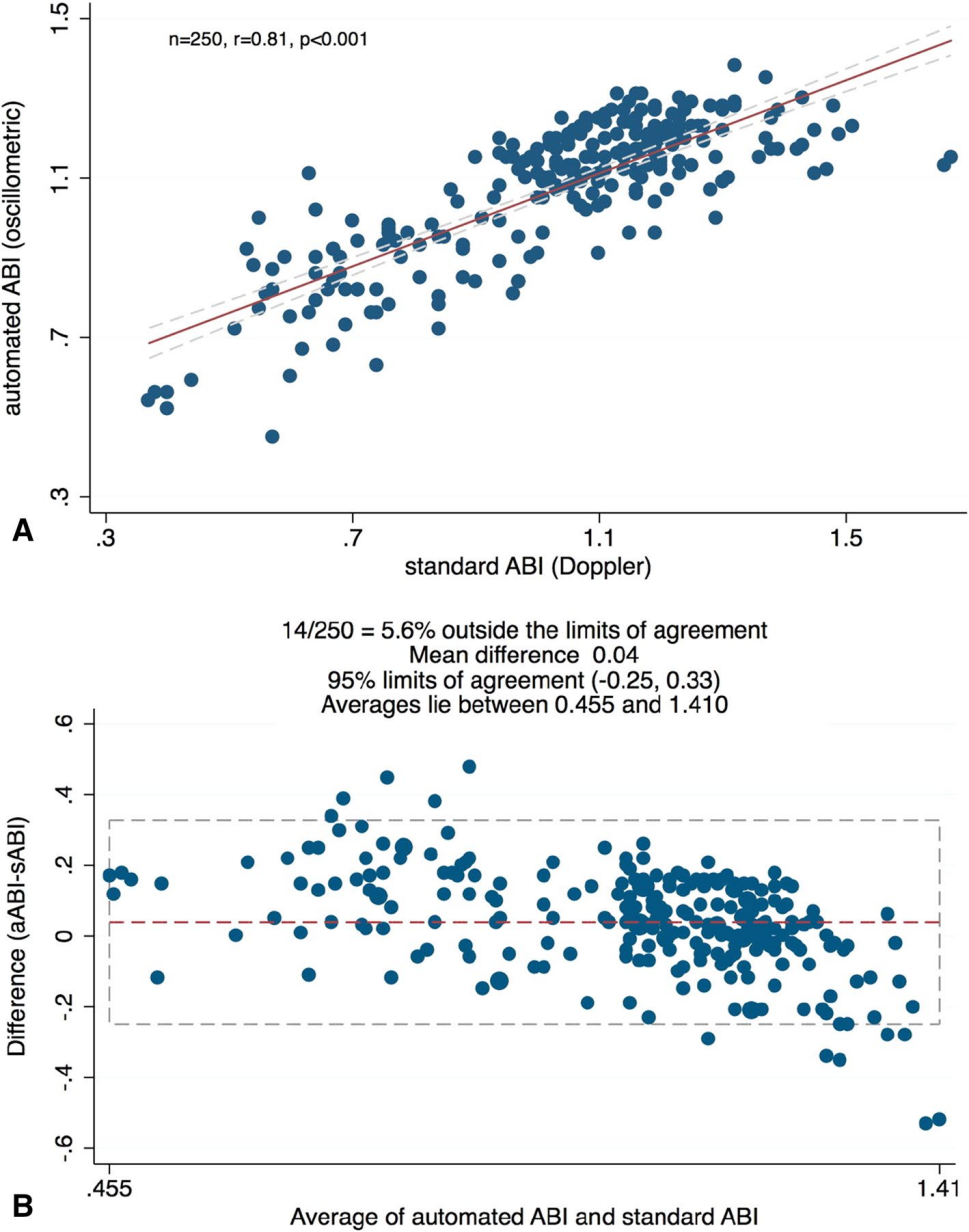
**a** A scatter plot illustrates a high correlation of *R* = 0.81 between standard Doppler ABI and automated oscillometric ABI measurements. Linear regression analysis reveals a probability *P* < .001. **b** Bland–Altman plot reveals high level of agreement between average and difference of the means of standard Doppler ABI and automated oscillometric ABI measurements

Figure 2a shows the correlation between the two ABI measurement modes. At *r* = 0.81 the agreement between sABI and aABI was highly significant (*P* < .001).

Before application of Bland–Altman analysis the distribution of the differences between the methods was inspected regarding normality. The differences were found to be normally distributed. Bland–Altman analysis performed on all 250 measurements indicates a mean difference of 0.04, and 95% limits of agreement between the two methods ranging from −0.25 to 0.33 (Fig. 2b).

### Additional investigations for patient subgroups

Since the prevalence of type 2 diabetes mellitus in the patient group provided for diabetes status-specific grouping of patients (a fact unforeseeable at the study’s planning stage) we became interested in a subgroup specific comparison of the two ABI measurement methods as described above.

**Fig. 3:**
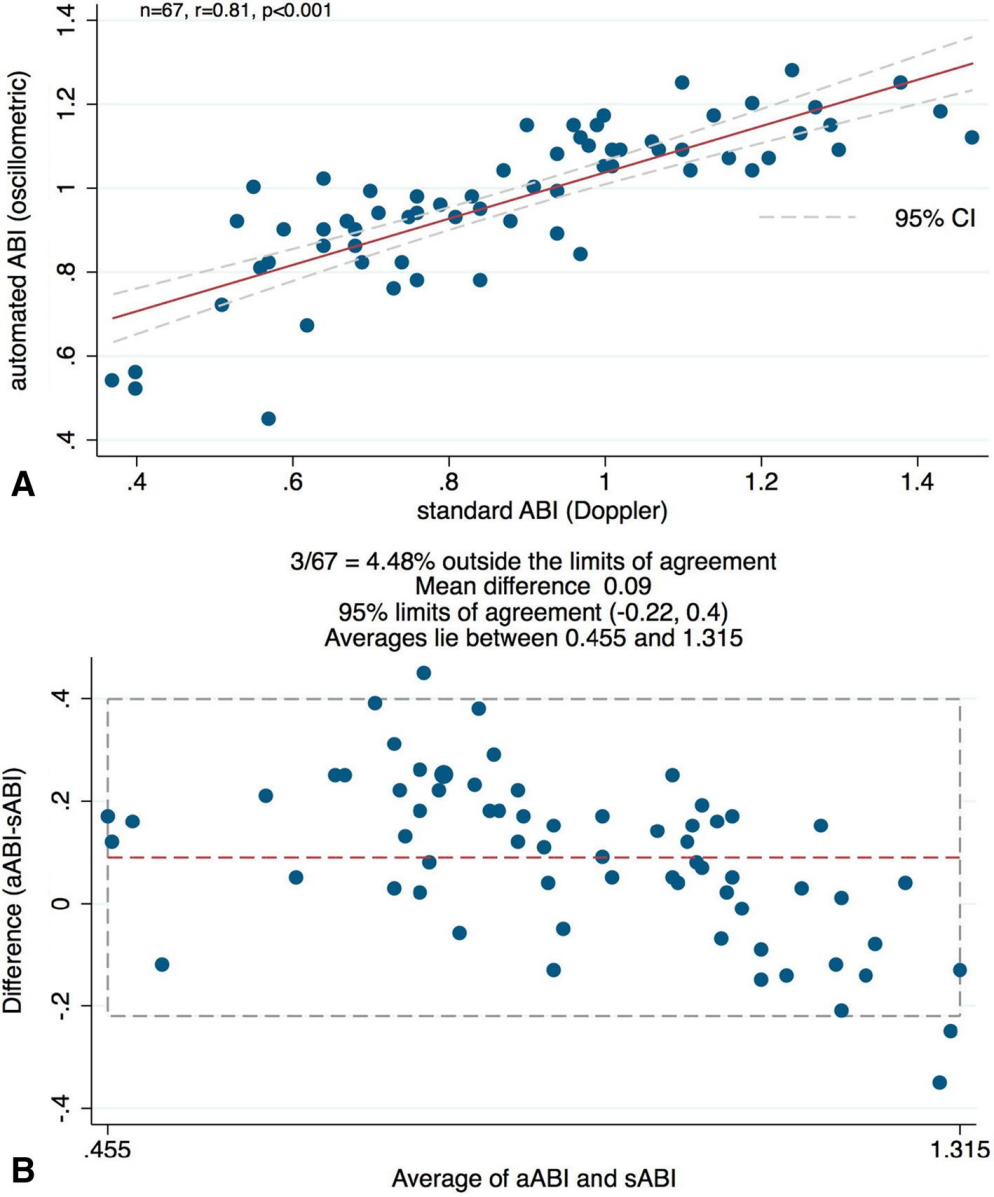
**a** A scatter plot illustrates a high correlation of *R* = 0.81 between standard Doppler ABI and automated oscillometric ABI measurements in the *diabetic* patients subgroup. Linear regression analysis reveals a probability *p* < .001. **b** Bland–Altman plot reveals a high level of agreement between average and difference of the means of standard Doppler ABI and automated oscillometric ABI measurements in the *diabetic* patients subgroup

**Fig. 4:**
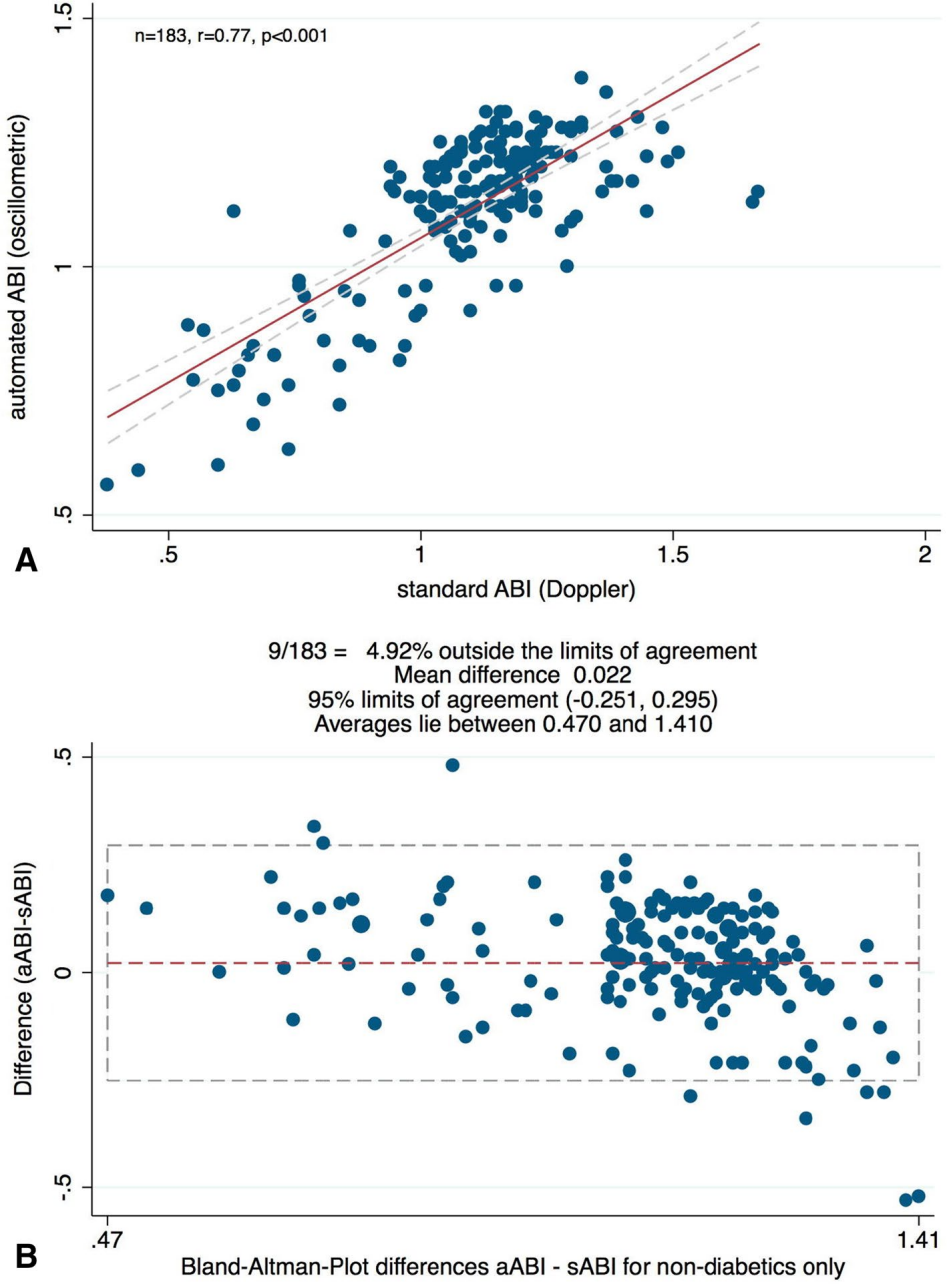
**a** A scatter plot illustrates a high correlation of *R* = 0.77 between standard Doppler ABI and automated oscillometric ABI measurements in the *non-diabetic* patients subgroup. Linear regression analysis reveals a probability *P* < .001. **b** Bland–Altman plot reveals a high level of agreement between average and difference of the means of standard Doppler ABI and automated oscillometric ABI measurements in the *non-diabetic* patients subgroup

**Fig. 5:**
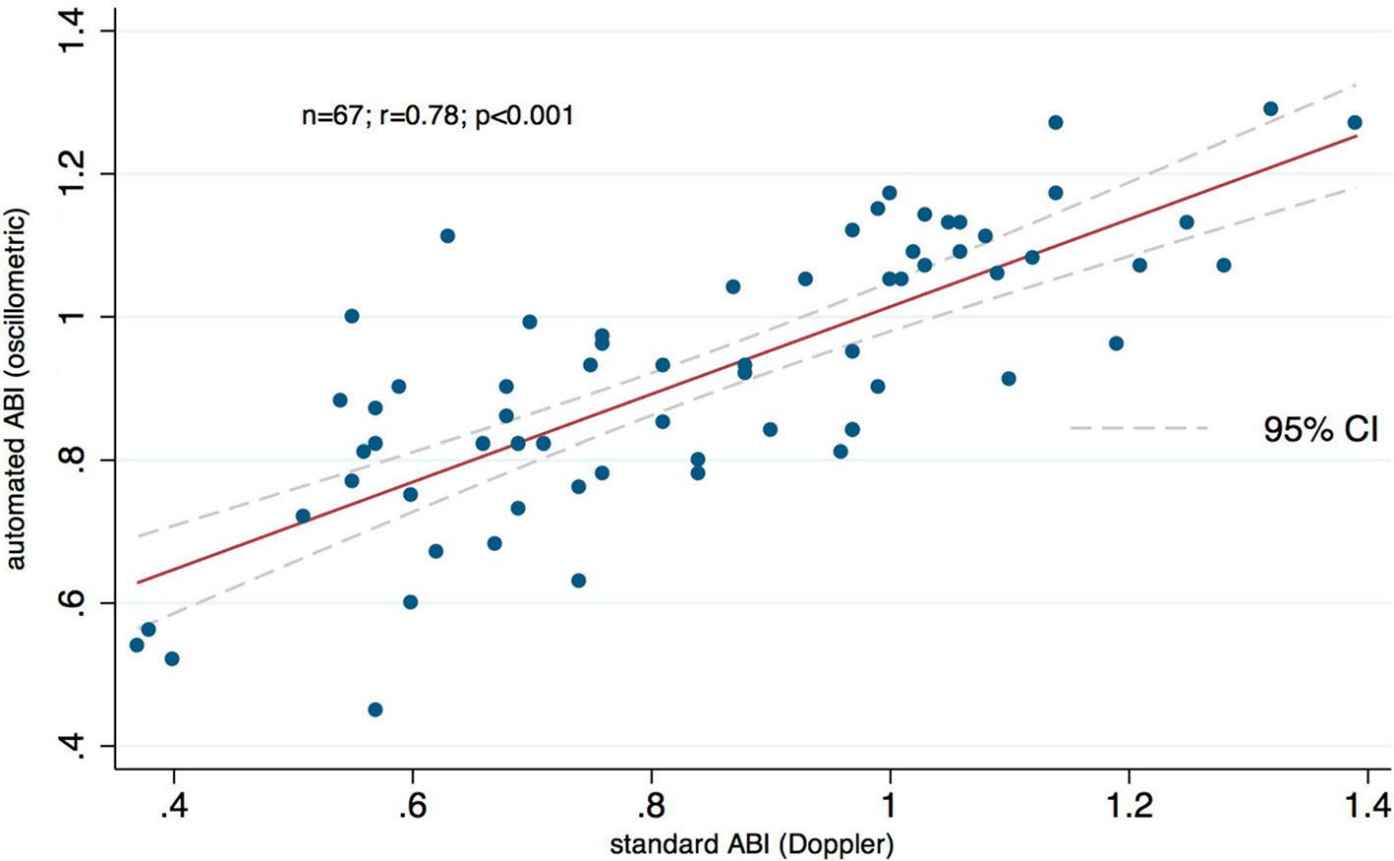
A scatter plot illustrates a high correlation of *R* = 0.78 between standard Doppler ABI and automated oscillometric ABI measurements in the subgroup of patients with symptomatic PAD before endovascular treatment. Linear regression analysis reveals a probability *P* < .001

When measurements were separated into diabetic and non-diabetic subgroups (20 subjects in symptomatic PAD group; 4 subjects in asymptomatic control group) in the respective values for bias and limits of agreements were 0.09 and −0.22 to 0.4 for diabetics, and 0.022 and −0.25 to 0.295 for non-diabetics, respectively (Figs. 3b, 4b). Pearson correlations between the two methods were *r* = 0.81 (*P* < .001) and *r* = 0.77 (*P* < .001) for diabetics and non-diabetics, respectively (Figs. 3a, 4a). Figure 5 illustrates the results of a subgroup of patients with symptomatic PAD (pain-free walking distance <200 m), who were scheduled for endovascular treatment. Pearson correlation coefficient between the two methods was *r* = 0.78 (*P* < .001), which highlights the power of automated oscillometric measurement even in patients with clinically relevant PAD and, therefore, only low ABI.

### Investigation of arterial stiffness index in comparison to flow-mediated dilatation (FMD)

Pre-intervention FMD and AS showed a borderline significant correlation, with *R* = 0.386, *P* = .043. No correlation was observed between post-interventional FMD and AS (*R* = −0.06. *P* = .76). FMD increased non-significantly by 0.15% from pre- to post-intervention (*t* = 1.57, *P* = .063).

### Change of arterial stiffness pre- to post-intervention

Conversely, the increase in AS from a pre-intervention mean of 504.2 to a post-intervention mean of 704.4 was highly significant (*t* = 3.82, *P* < .001), reflecting a mean increase by 51 ± 54.1%, with 82% of patients (23 of 28) witnessing an increase in AS.

## Discussion

Assessment of ankle-brachial index (ABI) is recommended for PAD screening by the current guidelines of the European Society of Hypertension and Cardiology. ABI acquisition is usually made by non-invasive measurement with a very high sensitivity and specificity and considered as the method of choice [21–23]. However, standard Doppler ABI measurement is time-consuming and requires specific skills resulting in a lower frequency of its acquisition in daily routine [4, 5]. Doppler measurements necessitate additional steps, e.g., pulse auscultation and supra-systolic compression combined with Doppler signal evaluation. Consistency analysis has already shown a very large variety for training of ABI acquisition and outcome measures [24]. Recent studies have evaluated automated oscillometric blood pressure monitors, which have the potential to reduce examination time. Davies et al. described automated measurements being significantly faster than Doppler measurements (7 min 55 s vs. 17 min 45 s; *p* < .01) [5]. Oscillometric and Doppler methods agree in terms of the ABI associations and differences as well as the diagnosis of PAD [25]. Oscillometric ABI assessment appears to be feasible, faster and accurate in clinical practice [26], while investigator-related intra-observer and inter-observer bias and error are reduced [7]. Simultaneous arm-leg measurements resulted in a smaller difference between the average oscillometric ABI value and the average Doppler ABI than did sequential measurements [4, 5]. Data suggest that automated ABI measurement by oscillometric blood pressure devices is a reliable and practical alternative to the conventional Doppler measurements for the detection of PAD. In case of erroneous or lack of oscillometric measurement the probability for PAD is very high [5]. A large meta-analysis revealed that oscillometric ABI determination is characterized by slightly higher ABI values. A possible explanation is a systematic error assessing Doppler ABI likely due to observer error caused by the delay between Doppler signal auscultation, viewing and recording the sphygmomanometer for arm and ankle. Additionally, the (more sensitive) modified ABI (which means using the lower instead of the higher ankle pressure) is not possible because the oscillometric method acquires the posterior and anterior (or dorsal pedal) tibial artery simultaneously [4]. The simultaneous measurements result in a significantly smaller ABI difference compared to sequential (Doppler ABI) assessment. Moreover, a clear advantage of simultaneous oscillometric assessment is prevention of random blood pressure variation [4, 5]. Since ABI measurements are acquired to rule out or suspect the presence of PAD—the latter case necessitating follow-up investigations or invasive treatment procedures about improved and maintained vessel integrity—correct assessment of PAD is the benchmark criterion to determine compatibility of ABI measurement methods. Therefore, the aim is to detect PAD at an early stage. Especially, in a population as it typically presents to a vascular health care provider, high discriminatory power in terms of secondary prevention is essential. Hence, the patients’ group included a majority of patients with diabetes mellitus. The control group consisted of PAD-free participants with a non-compromised, normal walking capacity. Incompressibility of vessels secondary to atherosclerotic calcification poses a challenge for performing ABI, as evidenced in the dramatically decreased sensitivity of ABI in diabetic patients. Which is why the additional information of PWV provided by an oscillometric device, together with the latter system’s recording of pulse pressure curves, contributes valuable information about the status of the vessels. Proper application of Bland–Altman analysis for evaluation of two different methods typically requires an *a priori* definition of the acceptable limits of agreement to support or reject interchangeability of measurement methods [20]. The results of our study reveal absence of significant difference in classification between the two tested methods. There is only a small bias and acceptable limits of agreement suggest the VascAssist device as a suitable substitute for Doppler-based measurement of ABI. Doppler-based ABI measurement is considered as the standard of choice. However, sequential measurement of blood pressure is used in the upper arms, the dorsal pedal and tibial arteries. Normal blood pressure fluctuations inevitably affect the results of the sABI calculation, which is determined as the ratio of pressures measured sequentially rather than simultaneously. Conversely, this inherent methodological flaw is not present, when using aABI as determined by VascAssist, because it provides simultaneous pressure recordings of arms and legs.

**Fig. 6:**
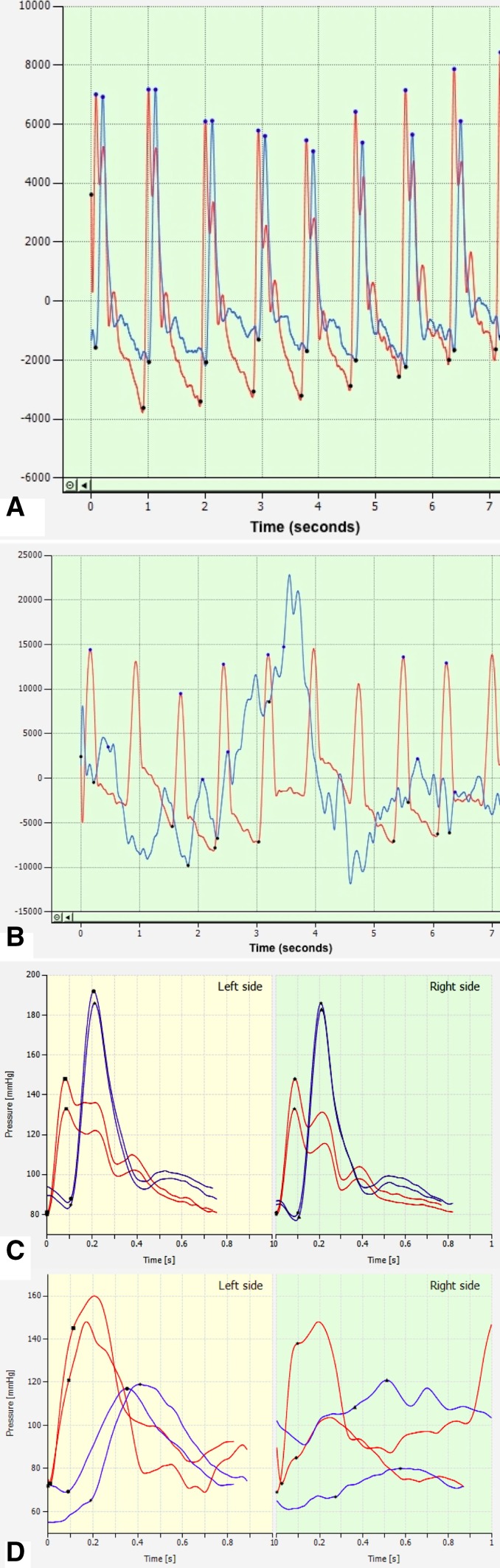
Illustrations of the automated oscillometric ABI measurement: **a** Automated oscillometric acquisition of brachial (*red*) and ankle (*blue*) pulse pressure curves in a healthy individual are characterized by clearly demarcated and identifiable curves with distinct systolic peaks. **b** Pulse pressure curve assessment in a patient suffering symptomatic PAD with reduced pain-free walking distance <75 m is characterized by chaotic deviations from the normal rhythmic pulsatility. **c** Data acquired by the VascAssist device processed by the VascViewer software averages ensembles of 15 heart cycles into one pressure tracing for analysis. The example shows a typical pressure wave in a healthy subject. **d** Corresponding to (**b**), data averaging over several heart cycles into one pressure tracing in patients with PAD results in disordered curve shapes

Increased AS is identified as an independent factor for cardiovascular mortality. It is typically associated with age, hypertension, diabetes, end-stage renal disease and peripheral arterial disease [27]. Central and peripheral arterial PWV are specific indicators and considered as the method of choice for the assessment of AS and the severity of peripheral vascular disease [28, 29]. ABI measurements narrow the diagnostic focus to the systolic peaks of the pulse pressure curves, thereby neglecting the wealth of information contained in the latter. As a consequence, PWA is considered as a standard test for peripheral arterial disease in clinical practice, as it is the case for ABI. Central and peripheral PWV are related to ankle-brachial pressure index [30, 31]. Depending on the number and/or severity of stenoses, the downstream pressure curves may change from rhythmic pulsatility to chaotic tracings. As it is evident in Fig. 6a the systolic peaks of oscillometric brachial (red tracing) and ankle pressure (blue tracing) curves are clearly demarcated and identifiable in a healthy individual. Conversely, the ankle tracings of a patient with severe PAD resulting in an impaired walking capacity <30 m (Fig. 6b) show chaotic deviations from the normal rhythmic pulsatility. The device used in this study averages 15 heart cycles into one pressure tracing for analysis (healthy subject in Fig. 6c, claudicant in Fig. 6d). It could be argued that this method of ABI measurement provides more reliable and repeatable results than standard manual measurements, which derive ABI at a single time point at which measured systolic pressure may or may not coincide with the time-averaged result of aABI measurement. Given a chaotic pressure curve, the degree of the two measurement methods is clearly left to chance. While the reliability of the ABI measurement can be verified by visual inspection of the pulse pressure tracing in the VascAssist, no such quality control is possible with standard ultrasound measurement.

Although a pathologic PWV has been described as preceding or accompanying cardiovascular diseases, evidence for such relationship in PAD is only less well documented [32, 33]. The present study provides strong data to support that clinically symptomatic PAD—diagnosed by invasive catheter angiography—is associated with a reduced PWV that increases after endovascular intervention. These data are in contrast to some other studies reporting on PWA in patients with PAD. But, in these studies definition and assessment of PAD with inclusion of arm and leg together with aortic large artery measurements on PWV was less well defined; i.e., ABI, the presence of claudication, or clinical evidence of arterial insufficiency was not required for the diagnosis of PAD [34, 35]. In contrast, our study is the first comparing functional vascular parameters including ABI, FMD, and AS with inclusion of the arms and legs together with aortic PWV assessment in PAD patients suffering from a more severe stage of atherosclerosis than those previously reported. Moreover, the present study was conducted on symptomatic PAD patient before and after an endovascular intervention. There was only a weak correlation between FMD and AS at baseline. This was probably just a chance finding since no correlation was observed following successful interventions. However, the strong and significant post-interventional increase of AS suggests that the improvement in arterial function, which is the objective of interventions in PAD patients, is obviously reflected in a change of AS. This finding warrants further investigations into the potential role of AS as an indicator of arterial health. Given its operator skill-independent mode of acquisition, AS may be an attractive robust alternative to error-prone FMD as a screening tool for arterial health. In addition it may serve as a control for successful PAD therapy by interventional means.

The limitations of the study consist of practical constraints on participant selection, which did not allow us to draw a random sample of the population in general or of the medical center’s patient population. Hence, the analysis of sensitivity, specificity, positive and negative predictive power may not apply to settings with substantially different proportions of diseased vs. healthy subjects. The conducted power analysis revealed 110 study subjects to be appropriate for adequate statistical hypothesis testing. The number of recruited subjects per group is limited (41 patients with symptomatic PAD and 69 voluntary healthy participants in control group free of PAD), and consequently the number of diabetics for sub-analyses.

## Conclusion

aABI measurement using the VascAssist device is interchangeable with manual ultrasound-based sABI measurement.

Arterial stiffness, as a result of total peripheral resistance and arterial compliance determined by PWV acquisition is a promising indicator of arterial health status and function, warranting further investigations for early detection of PAD in terms of secondary prevention.
